# Acoustic divergence in the communication of cryptic species of nocturnal primates (*Microcebus ssp*.)

**DOI:** 10.1186/1741-7007-6-19

**Published:** 2008-05-07

**Authors:** Pia Braune, Sabine Schmidt, Elke Zimmermann

**Affiliations:** 1 Institute of Zoology, University of Veterinary Medicine Hannover, Bünteweg 17, 30559 Hannover, Germany

## Abstract

**Background:**

A central question in evolutionary biology is how cryptic species maintain species cohesiveness in an area of sympatry. The coexistence of sympatrically living cryptic species requires the evolution of species-specific signalling and recognition systems. In nocturnal, dispersed living species, specific vocalisations have been suggested to act as an ideal premating isolation mechanism. We studied the structure and perception of male advertisement calls of three nocturnal, dispersed living mouse lemur species, the grey mouse lemur (*Microcebus murinus*), the golden brown mouse lemur (*M. ravelobensis*) and the Goodman's mouse lemur (*M. lehilahytsara*). The first two species occur sympatrically, the latter lives allopatrically to them.

**Results:**

A multi-parameter sound analysis revealed prominent differences in the frequency contour and in the duration of advertisement calls. To test whether mouse lemurs respond specifically to calls of the different species, we conducted a playback experiment with *M. murinus *from the field using advertisement calls and alarm whistle calls of all three species. Individuals responded significantly stronger to conspecific than to heterospecific advertisement calls but there were no differences in response behaviour towards statistically similar whistle calls of the three species. Furthermore, sympatric calls evoked weaker interest than allopatric advertisement calls.

**Conclusion:**

Our results provide the first evidence for a specific relevance of social calls for speciation in cryptic primates. They furthermore support that specific differences in signalling and recognition systems represent an efficient premating isolation mechanism contributing to species cohesiveness in sympatrically living species.

## Background

Cryptic species are closely related species which are morphologically similar, but differ genetically [1,2]. The recent development in molecular taxonomy and systematics has uncovered a rich diversity of cryptic species, in particular for nocturnal mammals [3-8].

A fundamental problem for sympatrically living, cryptic mammalian species is the coordination of reproduction between conspecifics in time and space, especially when individuals of a species forage solitarily. Under these circumstances mating partners do not only have to detect, localise and find each other, they also have to discriminate between conspecifics and remarkably similar heterospecifics. Current evolutionary theory [1,9-11] suggests that species cohesiveness in sympatry requires signalling and recognition systems acting as premating isolation mechanisms in order to avoid costly hybridisation. Sexual selection may cause a faster evolution of behavioural than of morphological traits [12,13]. While this theory has been supported by studies on the perception of the advertisement calls of crickets [14,15] and frogs [16,17], the songs of birds [18,19] and the social calls of bats [20], empirical data on other mammalian groups such as primates are still missing.

The Malagasy mouse lemurs, small nocturnal primates which inhabit the fine branch niche of forests provide an excellent model to explore the significance of vocal communication for species recognition and discrimination. Mouse lemurs have large mobile ears, exhibit a high auditory sensitivity [21], are highly vocal and show a rich repertoire of social calls [22-24] extending to the ultrasonic range, which is comparable to communication calls of microchiropteran bats, cetaceans, some rodents [25] and some frogs [26]. The call types of social calls in mouse lemurs are used in contexts of, for example, social cohesion (for example, trill), attention and alarm (for example, whistle) or agonistic situations (for example, tsak; cf. [22]).

At present 15 cryptic species are known which are difficult to distinguish in body characteristics [7,27-29]. In several areas two species occur sympatrically. Mouse lemurs live in a dispersed social system [30-32]. During the mating period, vocal activity in mates is enhanced [33,34], males actively search for oestrous females during the night and female choice may prevail [35,36].

We studied the structure of male advertisement calls of the grey, the golden brown and the Goodman's mouse lemur, formerly belonging to the rufous mouse lemur (*Microcebus rufus*) and their perception by the grey mouse lemur. These three species are genetically distinct from each other but share a large number of morphological features [7]. The first two species live sympatrically in dry deciduous forest of north-western Madagascar. The Goodman's mouse lemur, on the other hand, inhabits rainforest areas in eastern Madagascar, that is, it occurs allopatrically to the other studied species.

Male advertisement calls used in the reproductive context exhibited significant differences in call structure of allopatric mouse lemur species (the grey and the Goodman's mouse lemur) whereas alarm calls do not [37]. Until now, however, it is not known whether there are differences in vocal communication between sympatric species or whether mouse lemurs discriminate between social calls across species.

The present study gives the first account of the relevance of communication calls for species recognition and discrimination in cryptic primates in an area of sympatry combining a call structure analysis and playback experiments. Three questions were addressed:

1. To what extent do advertisement calls of sympatric cryptic mouse lemurs differ in structure?

2. Do mouse lemurs discriminate between advertisement calls of different species and do they show stronger discrimination between conspecific and sympatric than between conspecific and allopatric calls?

3. Do mouse lemurs discriminate between call types of different species which are irrelevant for species recognition in the reproductive context?

To answer these questions we recorded male advertisement calls of grey and golden brown mouse lemurs and measured several time and frequency parameters for an interspecific statistical comparison of the sympatrically living species. Per individual, we analysed 3 to 21 (median 5) calls and calculated individual median values for each acoustic parameter. On the basis of these values we conducted a Kruskal-Wallis analysis of variance (ANOVA) to test for species-specificity.

In addition we conducted playback experiments with 16 grey mouse lemurs from the field. Six categories of playback stimuli were presented during a single experimental session: conspecific male advertisement calls (referred to as conspecific advertisement), heterospecific male advertisement calls of the golden brown mouse lemur (referred to as sympatric advertisement), heterospecific male advertisement calls of the Goodman's mouse lemur (referred to as allopatric advertisement) and male whistle alarm calls [22,38] of all three species (referred to as conspecific whistle, sympatric whistle and allopatric whistle, respectively).

The behavioural responses of the tested individuals were classified into two different response categories: (1) no orientation, not involving any orientation response including no reaction, ear movement, interruption of activity or startle without turning towards the speaker and (2) orientation, including turning towards the speaker and approaching the speaker, sometimes accompanied by antiphonal vocalisation.

For statistical comparison of call categories, an individual-based analysis was conducted comparing individual response indices for all call categories of advertisement calls and short whistles, respectively. The individual response index towards a call category was defined by the number of orientation responses divided by all responses of an individual towards stimuli of the respective call category, that is

Individual response index = (Number of orientation responses of individual)/(Number of all responses of individual)

Friedman-ANOVA and Wilcoxon tests with a serial Bonferroni correction procedure [39] were performed for each call type.

## Results

### Interspecific comparison of advertisement calls

**Figure 1:**
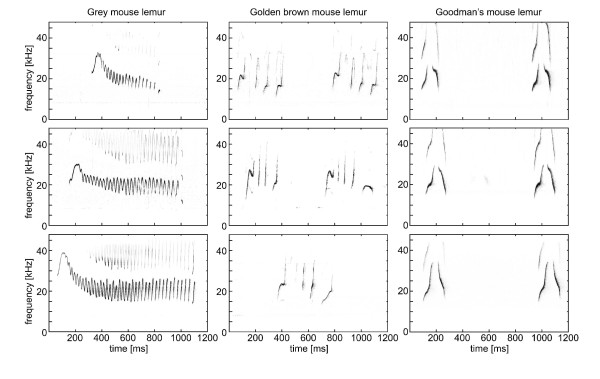
Representative sonagrams of advertisement calls emitted by three different individuals of the three studied mouse lemur species.

The frequency contour of the harmonically structured advertisement calls from the three species was remarkably different (Figure 1). The grey mouse lemur produced an acoustically complex, frequency modulated advertisement call with an upward frequency modulated sweep followed by a tail containing several sinusoidal modulations (sinusoidally frequency modulated call (SFM call)). The advertisement calls of the golden brown mouse lemur consisted of two to six generally upward frequency modulated components. Occasionally, a component contained a nearly constant frequency part and/or ended with a downward frequency modulated hook (upward frequency modulated with plateau-call (UFM call)). The Goodman's mouse lemur emitted a two-component call of relatively stereotypic structure with an upward followed by a downward modulated element separated by a short inter-element interval (inverse U-shaped call (IUS call)).

No measured frequency parameter showed any species specificity (Kruskal-Wallis test: *f*_0 _min: *H*_2 _= 3.470, *p *= 0.176; *f*_0 _max: *H*_2 _= 0.928, *p *= 0.629; *f*_0 _band: *H*_2 _= 2.566, *p *= 0.278; *N *= 14 for all tests; see Table 1), that is the absolute frequency ranges and the bandwidths of the advertisement calls of the three species were comparable. Call duration, however, differed significantly between the three species (Kruskal-Wallis test: *H*_2 _= 11.623, *p *= 0.003, *N *= 14). The calls of the grey mouse lemurs were the longest, those of the Goodman's mouse lemur the shortest and those of the golden brown mouse lemur took an intermediate position.

**Table 1:** Comparison of advertisement calls of three mouse lemur species.

Species	Note contour		Acoustic parameter	Median	Minimum	Maximum	25th percentile	75th percentile
***M. murinus***	SFM	Duration	(ms)	870	710	1,040	870	985
**(*N *= 5; *n *= 30)**		*f*_0 _min	(kHz)	12.30	12.00	13.95	12.20	13.80
		*f*_0 _max	(kHz)	35.90	34.90	37.80	35.60	36.40
		*f*_0 _band	(kHz)	23.10	20.90	25.20	21.60	23.20
***M. ravelobensis***	UFM	Duration	(ms)	375	360	430	365	405
**(*N *= 4; *n *= 39)**		*f*_0 _min	(kHz)	12.50	11.60	13.35	11.65	13.33
		*f*_0 _max	(kHz)	37.00	33.00	38.70	34.70	38.15
		*f*_0 _band	(kHz)	24.13	21.60	26.70	22.70	25.58
***M. lehilahytsara***	IUS	Duration	(ms)	135	120	160	135	150
**(*N *= 5; *n *= 20)**		*f*_0 _min	(kHz)	13.8	12.50	15.75	12.85	14.75
		*f*_0 _max	(kHz)	34.5	27.55	40.70	30.75	37.5
		*f*_0 _band	(kHz)	19.8	14.70	26.90	18.25	21.75

*N*, number of individuals; *n*, number of calls; SFM, sinusoidally frequency modulated call; UFM, upward frequency modulated with plateau call; IUS, inverse U-shaped call; for abbreviations of the acoustic parameters see Recordings and analysis of advertisement calls in Methods.

### Behavioural responses to advertisement and short whistle stimuli

**Figure 2:**
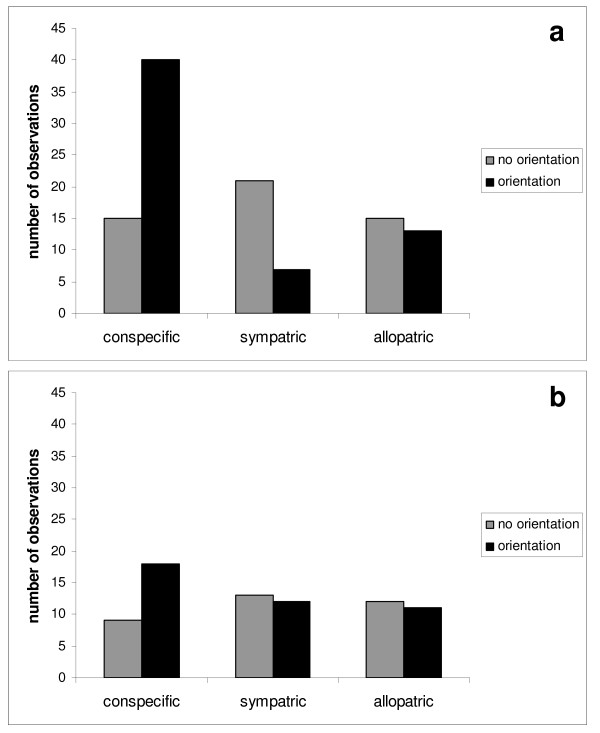
**Responses of grey mouse lemurs to playbacks**. Responses to playbacks of (a) conspecific (*M. murinus*), sympatric (*M. ravelobensis*) and allopatric (*M. lehilahytsara*) advertisement call stimuli and (b) short whistle stimuli. Please note that this figure summarises descriptive data for all individuals tested not analysed statistically.

In the 186 analysed responses the animals showed an orientation response in 101 cases, including turning towards the speaker on 85 occasions and approaching the speaker 16 times. In one of the latter cases a male additionally uttered an advertisement call after the presentation of a conspecific advertisement call. In the remaining 85 cases the animals showed no reaction to the stimuli in 48 cases, ear movement in 14, interruption of activity in 12 and startle without turning towards the speaker in 11 cases. An overview of the distribution of all analysed no orientation and orientation responses within the six call categories of all tested individuals is given in Figure 2.

Neither the sound pressure level nor the signal-to-noise ratio of stimuli had a significant effect on the stimulus response indices (Spearman rank correlations: sound pressure level, *r*_S _= 0.068, *N *= 22, *p *> 0.05; signal-to-noise ratio, *r*_S _= 0.411, *N *= 22, *p *> 0.05). In addition, response strength was independent of the presentation number of stimuli (Spearman rank correlation: *r*_S _= 0.378, *N *= 7, *p *> 0.05). This shows that inter-stimulus intervals were sufficient to avoid any habituation effects owing to the consecutive stimulus presentation design.

**Figure 3:**
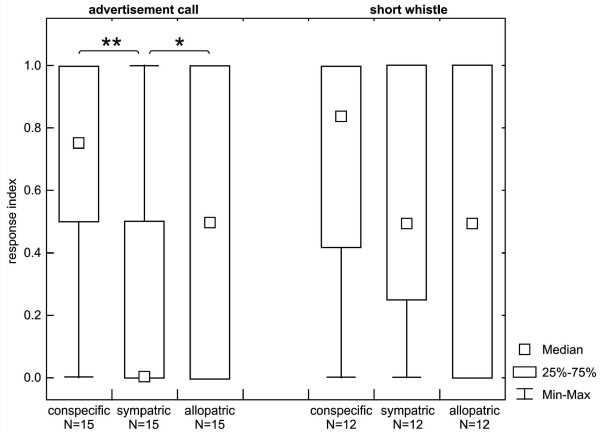
**Individual response indices for the different call categories**. *N *is the number of individuals, * indicates significant differences after serial Bonferroni correction (*p < 0.05; **p < 0.01).

Individual response indices revealed remarkable differences for conspecific, sympatric and allopatric stimuli (ANOVA, χ^2^_2 _= 12.298, *p *< 0.002, *N *= 15; see Figure 3). Thus, individuals reacted significantly more frequently with orientation responses towards conspecific than towards both sympatric and allopatric advertisement stimuli. This suggests a high interest of grey mouse lemurs for conspecifics advertisement stimuli compared with heterospecific advertisement stimuli. Furthermore, they responded significantly more frequently with orientation responses towards allopatric than towards sympatric advertisement stimuli (Wilcoxon signed-rank tests: conspecific-sympatric, *T *= 4.0, *N *= 15, *p *= 0.004; conspecific-allopatric, *T *= 15.0, *N *= 15, *p *= 0.060; sympatric-allopatric, *T *= 4.0, *N *= 15, *p *= 0.05; the conspecific-sympatric and sympatric-allopatric comparisons remained significant after serial Bonferroni correction).

In contrast, the individual-based analysis showed no significant differences in response strength towards all short whistle categories (ANOVA, χ^2^_2 _= 0.780, *N *= 12, *p *< 0.677; Wilcoxon signed-rank tests: conspecific-sympatric, *T *= 24.5, *N *= 12, *p *= 0.450; conspecific-allopatric, *T *= 15.0, *N *= 12, *p *= 0.374; sympatric-allopatric, *T *= 19.5, *N *= 12, *p *= 0.722; see Figure 3). These findings suggest that the grey mouse lemurs had no preference for any category of the short whistles.

## Discussion

The interspecific comparison of male advertisement calls of three mouse lemur species revealed structural differences as well as differences in response behaviour to playbacks. Both indicate a species-specific function of these calls. Conspecific calls evoked the strongest responses. Playback experiments furthermore suggest a different relevance of heterospecific advertisement calls with regard to sympatry or allopatry as sympatric calls evoked lower responses than allopatric calls. In contrast, no preference for any whistle call category was found.

### Species-specific structure in advertisement calls

The evolution of species-specific signals is driven by a trade-off between sensory system characteristics, predation, environment and mate choice criteria [40]. In the present study, all species used broadband, frequency modulated advertisement calls in the same frequency range. Broadband, frequency modulated signals provide advantages for sound localisation [41,42]. Uniformity in frequency range may be explained by similar morphological constraints [43] and similar predation pressure [44] for the three species studied.

On the other hand, we found species-specific frequency contours in the advertisement calls which play an important role in courtship and mating of mouse lemurs [34,35]. This divergence may reflect the high sexual selection pressure existing for advertisement calls [45]. Moreover, this divergence constitutes first evidence in primates for a behavioural trait evolving faster than morphological traits. The species-specific differences of advertisement calls could have evolved as an adaptation to transmission over long distances in different microhabitats as suggested for a number of different vertebrate taxa [46-48]. According to this habitat adaptation hypothesis [49], longer calls with short, rapidly repeated elements are favoured in more open habitats and shorter, slower modulated elements in denser vegetation structure [50].

In fact, the grey mouse lemur lives in dry deciduous forests and produces the longest call consisting of partially connected, rapidly repeated short elements. In contrast, the Goodman's mouse lemur, which occurs in rainforest areas characterised by dense vegetation emits the shortest call consisting of two longer elements only. Accordingly, shorter calls with separate more slowly modulated elements might have been the primary adaptation to the rainforest habitat. The call of the golden brown mouse lemur, which lives sympatrically with the grey mouse lemur, but is genetically closer related to the Goodman's mouse lemur [4], takes an intermediary position. The migration of the golden brown mouse lemur from rainforests into more open habitats [7,51-53] may have driven selection towards longer calls with more rapidly modulated elements. Thus, our results support the habitat adaptation hypothesis for this call type.

### Species-specific call recognition

Structural differences in advertisement calls of the three species do not necessarily represent evidence for the use of these calls in conspecific recognition. We showed in this study that grey mouse lemurs responded similarly towards the structurally similar whistle calls of the three species. This is not surprising as they occur in alarm situations [38] for which calls of a similar structure is used by a broad range of species and yield the same anti-predator responses [44]. As these calls are not counter-selected by sexual selection this trait remains stable.

In contrast, species-specific recognition of advertisement calls plays an important role for reproduction in cryptic and dispersed living species where females and males have to find each other for courtship and mating [12]. Thus, a positive response behaviour towards heterospecific calls would have a negative impact on the fitness of individuals as they would risk costly hybridisation. Our playback experiment confirms the above hypothesis for the first time in dispersed living primates: conspecific calls caused stronger interest than heterospecific calls. This response behaviour was not due to differences in stimulus quality. Therefore, an influence of sound quality on the response behaviour does not account for the differential responses to the different stimulus classes.

We found more pronounced differences in the perception of conspecific versus sympatric than versus allopatric calls. Comparable differences in perception have been reported from a wide range of species [15,16,54,55]. Character displacement [56] describes a pattern of greater divergence of an isolating trait in areas of sympatry between closely related taxa than in areas of allopatry [57]. As a result of selection against hybrids this mechanism may cause species-specificity in recognition systems (cf. [16] for frogs). This explanation may also account for our data (however, see [58] for birds). Alternatively, the observed differences in the perception of sympatric and allopatric advertisement calls could be a result of different exposure of the grey mouse lemurs to these calls. The grey mouse lemurs in our experiments would have been habituated to the sympatric calls over a long time and the increased attention towards the allopatric calls compared to the sympatric calls may represent a novelty effect [59].

## Conclusion

This study provides the first evidence in primates for specific acoustic divergence in the communication of cryptic species living in sympatry. Advertisement calls of sympatric species essential in the reproductive context differed distinctly in their structure whereas whistle calls irrelevant for reproduction did not. On the perception side mouse lemurs discriminated between the species-specific advertisement calls and preferred conspecific to sympatric calls. Thus, our data support the evolutionary hypothesis that species cohesiveness in sympatry has led to specific divergence in signalling and recognition systems in order to avoid costly hybridisation.

## Methods

### Recording and analysis of advertisement calls

Male advertisement calls were recorded in the presence of oestrous females [23,60]. The calls of five grey mouse lemurs and four golden brown mouse lemurs from the Ampijoroa population and five Goodman's mouse lemurs from the Hannover laboratory colony (originating from Andasibe, Madagascar) were recorded using two different media: a 1/2'' Bruel & Kjaer microphone (type 4133) with preamplifiers (type 2669 and 2619) connected to a NAGRA IV-SJ tape recorder (Kudelski SA, Switzerland) equipped with BASF tapes (ferro LH HiFi TP18, 38 cm/s); or a bat detector (U30, Ultrasound Advice) connected via a filter/control unit (Pettersson) to a high-speed A/D-card (DAS 16/330, Computerboards, Inc.) in a laptop (Compaq Armada) using the recording software BatSoundPro 3.0. All advertisement calls were recorded from caged animals at a distance of about 1 m. The vocalisations recorded with the NAGRA tape recorder were replayed at half speed and digitised with a sampling rate of 44.1 kHz (16 bit). We analysed all calls with BatSoundPro 3.0, using a fast Fourier transform (FFT) size of 512 and a Hanning window for spectrograms. For each advertisement call, we measured its duration, minimum (*f*_0 _min) and maximum (*f*_0 _max) frequency of the fundamental and calculated the bandwidth of the fundamental (*f*_0 _band = *f*_0 _max - *f*_0 _min).

Statistics were made using Statistica 6.0 (StatSoft Inc.), the level of significance was 0.05 for all statistical tests.

### Playback experiments

Playback experiments were conducted in the Ankarafantsika National Park (16°19'S, 46°48'E), about 110 km south-east of Mahajanga, Madagascar during the dry season from September to October 2000 and from July to October 2001 covering the mating period of the mouse lemurs. They were performed in a part of the dry deciduous forest where the grey and the golden brown mouse lemur occur sympatrically.

Sixteen (13 males, 3 females) grey mouse lemurs were subjects of our playback experiments. The experiments were conducted under temporary captivity conditions in the field. A stationary setup under controlled conditions was necessary because mouse lemurs communicate in the ultrasonic range which requires special playback and recording equipment. To test for differences in the perception of sympatric and allopatric calls, we needed animals from the field which were experienced with their sympatric species.

The intervention on the individual and population level by the experimental study was reduced to a minimum by the following procedure: we trapped the animals using Sherman Live Traps (HB Sherman Traps, Inc., Tallahassee, Florida) by setting them in the late afternoon in trees and bushes [61]. Traps were equipped with pieces of banana providing sufficient food and water supply for a night. Mouse lemurs have adaptations to dry conditions as they are able to gain water by metabolising brown fat tissue [62]. Traps were checked and collected in the early mornings. Captured mouse lemurs were brought to the observation cages in their traps. Individually identified animals were placed singly in cages of 1.2 m × 1 m × 0.5 m installed between bushy vegetation. These observation cages were equipped with a bamboo trunk as a nesting place, several branches and a bowl filled with water. The animals were fed with pieces of banana daily and they caught insects that entered the cages. The animals were housed between three and five nights and released afterwards at their capture point at sunset. The difference in caging times was due to the different habituation time individuals needed to behave normally in the presence of an observer and the number of playback sessions in which they performed (explained below). No individual which took part in the experiments showed any abnormal behaviour or injuries during the experiments or while housed in the cage. All mouse lemurs ate normally, moved in the cage and showed a normal day-night rhythm.

Due to the fact that mouse lemurs are seasonal breeders [63], it was guaranteed that no female was lactating or even advanced in pregnancy. After their release, many of the tested mouse lemurs were trapped again in their previous home range: some after several days, others also in the following year, that is, the location of trapping was not avoided and trapping had no negative consequences for the individuals. In addition, former studies showed that trapping as applied in our study had no adverse effects on mortality or other aspects of behaviour [64,65] and did not have a lasting effect on the population structure of grey mouse lemurs in our study area [66-69].

For the playback experiment a playback stimulus consisted of one call for the categories conspecific and sympatric advertisement, two calls for the category allopatric advertisement and three calls for the three whistle categories, respectively. By using this setup we accounted for the different duration and repetition rates of male advertisement calls and short whistles from the different species. We used two different advertisement stimuli from each of four conspecific males, two from the Hannover population and two from a Madagascar population. In addition, two different stimuli from each of two sympatric and allopatric males were taken. As whistle stimuli, we used two short whistles each of two males of the grey, two males of the golden brown and one male of the Goodman's mouse lemur.

With these stimuli, we produced a playback tape based on the original analogue recordings from the NAGRA tape recorder. To minimise background noise the stimuli were highpass filtered at a frequency of 7 to 15 kHz depending on the minimum frequency of the call. The playback tape was started at a random position using a NAGRA IV-SJ tape recorder (Kudelski SA, Switzerland), a custom-made amplifier and a speaker (Leaf Tweeter EAS-10Th400A). Stimuli ranged between 70.5 and 83.0 dB sound pressure level at a distance of 1 m (RMS, Bruel & Kjaer Measuring Amplifier Type 2610), that is, sound pressure levels corresponded to the naturally occurring ranges. The loudspeaker was placed between 0.6 and 0.8 m above the ground at a distance of about 0.5 m from the cage to ensure a sufficient presentation quality of the highly directional ultrasonic calls at any position in the cage.

In each playback session seven different stimuli were played back in a random order: two of the category conspecific advertisement (one each of the two different populations), and one stimulus each of the other five categories. To avoid a habituation to playback stimuli, the inter-stimulus interval was kept between 1 and 10 minutes. Each individual took part in one to three playback sessions in which each of them received every stimulus only once.

Behavioural responses to playback stimuli were observed at a distance of about 5 m from the observation cage using a headlamp and binoculars and reported to a dictaphone for further analysis. We recorded the behavioural responses belonging to the categories 'orientation' and 'no orientation' within 10 seconds just after the onset of a stimulus. In all cases, response behaviour had finished within this period.

Cases were excluded in which animals were not visible to the observer because they went into their bamboo trunk or were hidden by cage enrichment. We were able to analyse 186 responses to playback stimuli. The frequencies of no orientation and orientation responses were determined per stimulus and per individual, respectively. We recorded 5 to 13 (median 8) responses for each stimulus. Each individual contributed between 3 and 20 responses (median 11.5). The behavioural responses were counted for the respective response categories and visualised within each call category.

We conducted Spearman rank correlations to exclude the effects of stimulus quality by correlating the response indices of all stimuli for which we saw behavioural responses (*N *= 22) with their sound pressure level and their signal-to-noise ratio, respectively. A stimulus response index was defined by the number of orientation responses divided by all responses towards a stimulus. To make sure that the consecutive presentation of playback stimuli resulted in independent responses we conducted a Spearman rank correlation for the response indices with the order of stimulus presentation. The order response index was defined by the order number of the orientation responses divided by all responses for the respective presentation number.

Furthermore, to test for habituation effects we analysed the response strength towards the first and the second stimulus of the two conspecific advertisement stimuli during a given playback session. A chi-square test revealed that the distribution of no orientation and orientation responses did not differ significantly between the first and the second conspecific advertisement stimulus (chi-square-test: χ^2^_1 _= 0.149, *p *= 0.7). Therefore, responses of an individual towards conspecific advertisement stimuli were averaged for further analysis.

## Authors' contributions

PB participated in the design of the study, conducted the experiments, performed the statistical analysis and prepared the manuscript. SS mentored the study, contributed to the technical design of the study and the preparation of the manuscript. EZ initiated, financed, mentored and contributed to the design of the study and the preparation of the manuscript. All authors read and approved the final manuscript.
